# Does one size fit all? A qualitative study about the need for individualized information transfer for orthognathic patients

**DOI:** 10.1186/s13005-022-00321-6

**Published:** 2022-06-30

**Authors:** Isabelle Graf, Anna Enders, Ute Karbach, Tatjana Mihailovic, Teresa Kruse, Melanie Pollklas, Karolin Höfer, Joachim Zöller, Bert Braumann

**Affiliations:** 1 Department of Orthodontics, Faculty of Medicine and University Hospital of Cologne, University of Cologne, Kerpener Str. 32, 50931 Cologne, Germany; 2 Department for Research and Quality Management, Federal Centre for Health Education (BZgA), Cologne, Germany; 3 Institute of Medical Sociology, Health Services Research and Rehabilitation Science, Faculties of Medicine and Human Sciences, University of Cologne, Cologne, Germany; 4 Clinical Psychology and Psychotherapy, University of Cologne, Cologne, Germany; 5 Department of Operative Dentistry and Periodontology, Faculty of Medicine and University Hospital of Cologne, University of Cologne, Cologne, Germany; 6 Department of Dental Surgery and Oral, Maxillofacial and Plastic Facial Surgery, Faculty of Medicine and University Hospital of Cologne, University of Cologne, Cologne, Germany

**Keywords:** Information needs, Orthodontics, Orthognathic surgery, Qualitative research

## Abstract

**Aims:**

For any orthodontic-orthognathic treatment, it is crucial that patients are provided with enough and proper information in order to make evidence-based decisions- not only prior to treatment start, but also throughout the course of therapy. Thus, the objectives of this qualitative study were to identify information needs of patients undergoing combined orthodontic-orthognathic treatment. Additionally, professionals’ perspectives were evaluated.

**Methods:**

A qualitative research approach was chosen in order to determine crucial aspects of information needs before and throughout treatment. With respect to a purposive sampling strategy and thematic saturation, we conducted ten semi-structured interviews with patients who had finished their orthodontic-orthognathic surgery treatments (five women, five men; being 21 to 34 years old). The indications for the combination treatment were severe skeletal Class IIs to Class IIIs with various vertical and transverse discrepancies. In addition, a multidisciplinary focus-group with six professionals from the maxillofacial surgery and orthodontic department (three women, three men; being 30 to 38 years old) helped to reflect about the experts’ point of views.

After transcription, data was categorized and analyzed by Mayring’s content analysis.

**Results:**

We identified three key themes. During this analysis, we focused on theme (1) ‘information transfer’ with its corresponding categories ‘information needs’ – depending on different treatment stages –, ‘source of information’ and ‘doctor-patient-communication’. The affected patients ranked individualized patient information and empathetic doctor-patient-communication high.

This was mostly in line with the professionals’ point of view. Verbal communication was seen as being the best way to communicate throughout treatment. The role of the internet as a source of information was seen diversely.

**Conclusion:**

This qualitative study highlights the need for individualized patient information and reveals both met and unmet information needs by patients. Although evidence-based written information is highly necessary for orthognathic patients and their families alike, it cannot replace an empathetic way of direct verbal doctor-patient-communication. It seems crucial to give specific individualized information at different treatment stages, starting at a thoroughly interdisciplinary screening at the very beginning.

## Introduction

Combined orthodontic-orthognathic surgery treatments are complex interventions. It is crucial that patients with dentofacial deformities are provided with sufficient and proper information in order to make evidence-based decisions – not only prior to treatment start, but also throughout the ongoing treatment. Adequate patient information and shared decision-making is one of the major keys to patient satisfaction and is often regarded as the foundation of overall treatment success [1–7]. Combined orthodontic-orthognathic surgery treatments involve *elective* surgeries, so ample patient information is very important in order to understand treatment options, surgical procedures and their possible side effects [8]. But what is *sufficient* patient information? How much and what kind of information do our patients seek for? Is this generalizable? The individual information need might also depend on the specific psychological profile of the corresponding patient. Orthodontists and/or surgeons must pay close attention to patients who might exhibit specific psychological disorders or abnormalities at first consultation [9, 10]. This is a challenging task, especially because orthodontists and maxillofacial surgeons do not have psychological expertise regularly.

Researchers found, that even though professionals thought that they had given their patients enough and proper information about their upcoming treatment at its beginning, patients seemed to had forgotten about these details, especially when revolving around psychosocial problems [11]. On the other hand, Witt and Bartsch showed that it was more likely for orthodontic patients to remember verbal information when possible problems like side effects or treatment risks were involved [12]. Although verbal doctor-patient-communication and an empathetic way to communicate seem to be highly important for patients, some authors would not abstain from supplementary visual tools [13, 14]. In addition to that, written information about combined orthodontic-orthognathic treatment and the potential side effects is a common tool of information transfer. Another way for obtaining information is the internet. Yet, researchers highlighted that the correctness and validity of internet-based information might be flawed and the amount of somewhat unfiltered data overwhelming. Social media platforms like YouTube, Twitter and Instagram frequently provide specific and biased information of one’s personal treatment journey that is not necessarily generalizable [6, 15, 16].

In general, qualitative research methods are renowned for their potential to illustrate opinions, feelings and experiences, yet they have not been intensely used in the past [17]. Qualitative research can be regarded as a naturalistic approach to describe someone’s perspectives, feelings, thoughts and experiences [8]. Among others, common qualitative research methods in healthcare are interviews and focus groups [18]. On the one hand, researchers might get a detailed individual insight of patients’ thoughts and feelings through potentially time-consuming interviews, while, on the other hand, it can be regarded as a major advantage of focus-groups, that researchers can gather multiple viewpoints in a short period of time. Qualitative research methods frequently involve certain sampling strategies, which differ from the common ones within quantitative research: One of these strategies is the approach of purposive sampling [19], which was used in the present research project.

Although some international qualitative studies in the field of orthognathic surgery already exist [6, 8, 9, 20, 21], there is no publication that includes both patients’ *and* professionals’ point of views. In addition, one must keep in mind, that patients’ (and professionals’) thoughts and beliefs might change over the years – due to social, environmental and/or educational shifts in society, for example. Thus, it seems crucial to constantly reflect about our patients’ feelings and opinions, especially with regard to elective surgical procedures, so to be able to provide patient-centered care. Therefore, the objectives of this qualitative study were to identify information needs of patients undergoing combined orthodontic-orthognathic treatment. In addition to that, professionals’ perspectives were evaluated.

## Subjects and methods

This qualitative study was approved by the Committee of Ethics of the University Hospital of Cologne (#15–280). To investigate patients’ perspectives, adult patients were asked to participate in this research project at the end of their combined orthodontic-orthognathic surgery treatment at the said Hospital. Once they agreed to participate, an interview was conducted during their very last retention appointment at the orthodontic department. The time span between orthognathic surgery and recruitment was supposed to be at least 1 year, considering that general patient satisfaction and perception of treatment might be influenced directly after surgical procedures due to persistent side-effects [8]. Exclusion criteria were patients with severe systemic diseases, immunosuppression, oro- and/or craniofacial clefts and/or syndromes as well as patients with a trauma history. In general, qualitative studies use inductive ways to sample study participants. Thus, the technique of purposive sampling was chosen in order to potentially include the full range of possibly affected patients (e.g. female and male Class IIs and Class IIIs with various vertical discrepancies) and be able to make conceptual generalization, rather than statistical one [19, 22]. Study participants were not only picked out in order to just test the key study questions and confirm expected theories, but also to possibly contradict the already existing results in order to get the full grasp of patients’ perspectives on information needs during the course of combined orthodontic-orthognathic surgery. We followed the strategy of thematic saturation, so at the point when there was no novel aspect deriving from the data obtained by the interviews according to redundancy check, we assumed thematic saturation to be reached and no more study participant was included. All interviews were conducted by the same interviewer who had not been actively involved in participants’ treatments in order to minimize interviewer bias. The same setting was chosen for all interviews, in agreement with every participant: a quiet, non-clinical room within the University of Cologne. Ten patients, five women and five men, were finally included, representing a heterogeneous sample with varying initial indications to treat (Table 1).

**Table 1:** Characteristics of patients interviewed

	Female	Male
	*n* = 5	*n* = 5
Mean age (y)	24.6 (±4.8)	26.6 (±4.8)
Indication to treat		
Sagittal discrepancy (total)	5	5
Skeletal class II	1	1
Skeletal class III	4	4
Vertical discrepancy (total)	4	2
Skeletal openbite	3	1
Skeletal deepbite	1	1
Transverse discrepancy		
Crossbite	3	3

As the interviews were chosen to be semi-structured, we developed an interview guide with 16 open key questions based on knowledge and assumptions from previous research. This occurred in cooperation with the Institute of Medical Sociology, Health Services Research and Rehabilitation Science of the University of Cologne.

To find out about the professionals’ opinions and thoughts about our research questions, both departments – orthodontic and maxillofacial surgery – took part in the study. Two surgeons (one woman, one man) and four orthodontists (two women, two men) between 30 and 38 years of age (mean age 34.2) joined in the focus-group meeting which was arranged in the same room of the University of Cologne as described before. These professionals had not necessarily been part of the treatments of those patients who were recruited for this study. The same person conducted all interviews and was the moderator of the focus-group. The conceptual focus-group guideline with 16 open questions was coordinated with the applied interview guide. Like all interviews, the discussion of the focus-group was digitally recorded. Interviews and focus-group dialogues lasted from 20 to 60 minutes.

### Data collection and analysis

All data was transcribed verbatim by two investigators. Both transcripts were then discussed and a consensus was reached, if necessary. Data was qualitatively analyzed by using Mayring’s content analysis [23], establishing a category system that represented optimally the content of both interviews and focus-group discussion. Accordingly, two researchers screened independently parts of the text material to pre-define potential codes and categories, while codes that matched thematically were combined to categories. After analyzing all data, this preliminary category system was refined. Overarching themes were those which combined several categories. The category system of both experimenters was affirmed by the Institute of Medical Sociology, Health Services Research and Rehabilitation Science of the University of Cologne. In addition to that, word clouds were created in order to visualize results regarding the source of information. Word clouds generally help to make the reader see results instantly. Words with a larger font size represent more frequently counted words than those with a smaller font size. Some words were combined for better understanding (e.g. “whatisright” or “toomuchinformation”). Maxqda Software was used for all above-mentioned analyses.

## Results

**Fig. 1:**
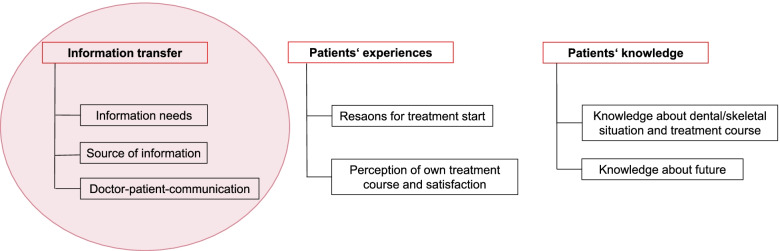
Category system according to Mayring

We identified three overarching themes (1) ‘information transfer’, (2) ‘patients’ experiences’ and (3) ‘patients’ knowledge’ as well as seven categories: ‘information needs’, ‘source of information’ and ‘doctor-patient-communication’ within (1); ‘reason for treatment start’ and ‘perception of own treatment course/satisfaction’ within (2); ‘knowledge about dental/skeletal situation and treatment course’ and ‘knowledge about future’ within (3) (Fig. 1). In the course of this research project, we focused on theme (1) ‘information transfer’ and the corresponding categories ‘information needs’, ‘source of information’ and ‘doctor-patient-communication’.

**Fig. 2:**
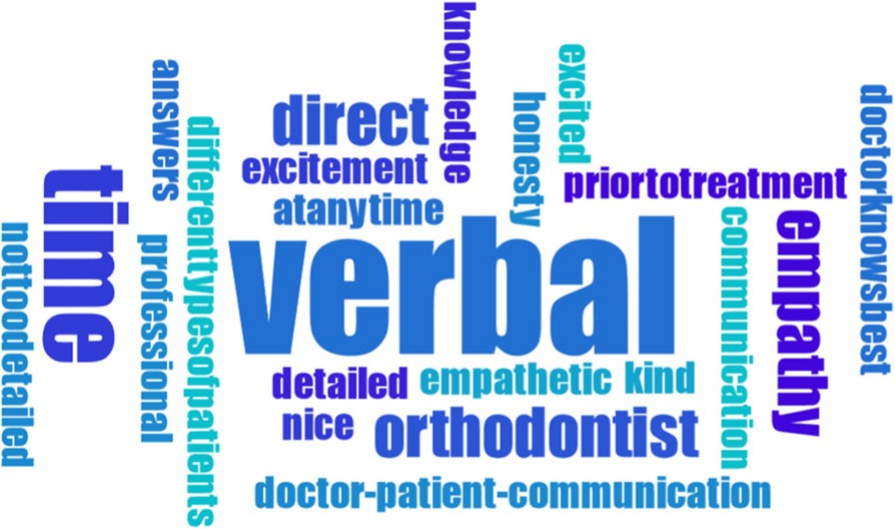
Word cloud about *verbal* information transfer

**Fig. 3:**
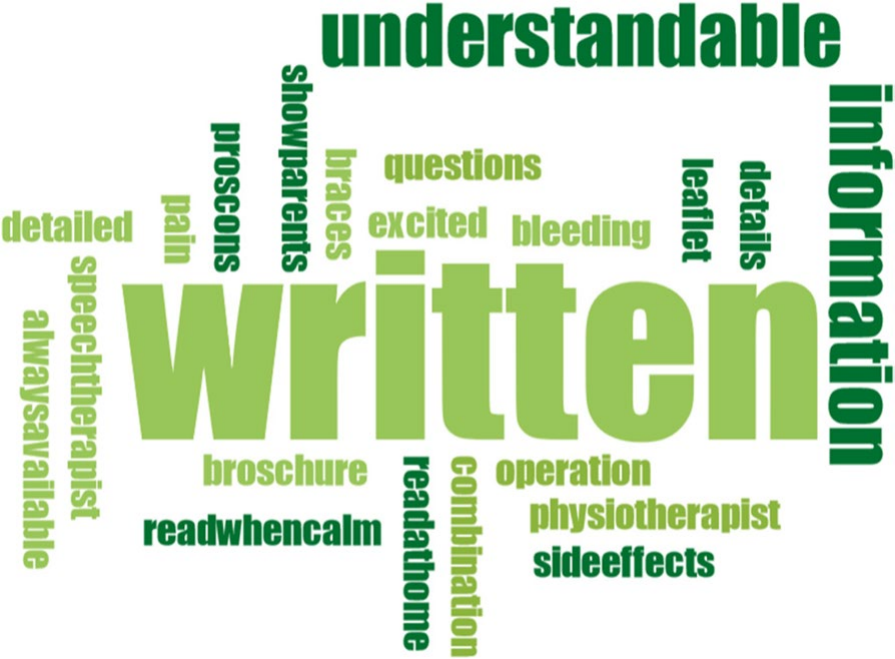
Word cloud about *written* information transfer

**Fig. 4:**
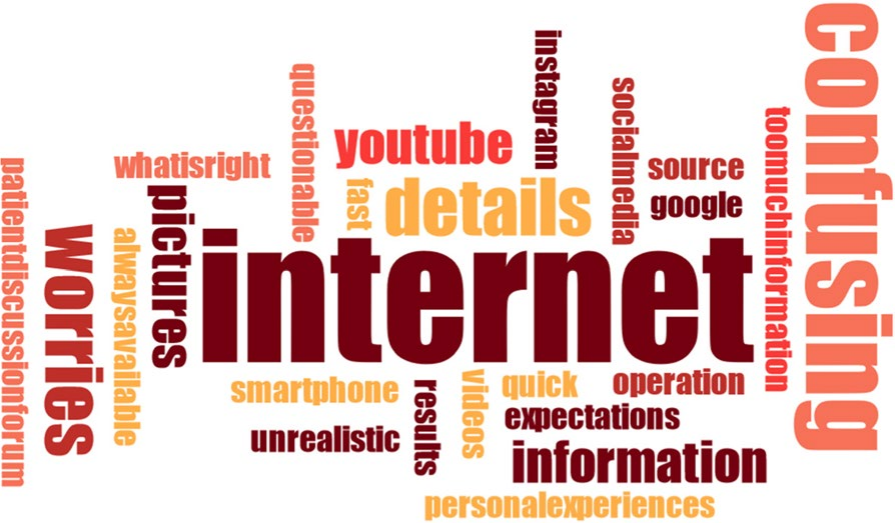
Word cloud about the *internet* as a source of information

In the following, a differentiation between (A) patients’ perspectives and (B) professionals’ perspectives is presented. Furthermore, all data from (A) and (B) regarding the ‘source of information’ was combined – subdivided into *verbal*, *written* and *internet* – and visualized through word clouds (Figs. 2, 3 and 4).

### (A) Patients’ perspectives

We found differing needs for information depending on the treatment stages in the course of orthognathic treatment. Therefore, we differentiated according to the stages ‘prior to treatment uptake’, ‘during orthodontic treatment’ and ‘prior to or during the stay at the hospital’. These were recurring crucial stages within the category ‘information needs’.

#### Information needs prior to treatment uptake

Patients seemed to want a broad overview of the planned treatment steps and enough time to think about the given information. Some mentioned the need for understandable treatment information prior to treatment uptake:

- Well, it’s most important to me that I know what the doctor plans to do and, ehm, and tries to explain that to me in a nutshell, mustn’t be detailed (pause) understandable!

Interestingly, there was also the clear wish for not too much information at treatment start.

- After going through all this, I’d say that I’m happy you guys didn’t tell me all of it directly, because I might have backed down then. That’s possible, I guess, because it sounds so horrible at that moment and you think to yourself ‚Oh my god!‘and well, it’s ok that it all came down as it has, because otherwise I would have been worried even more (frowns).

#### Information needs during orthodontic treatment

During treatment, patients emphasized the importance of personal and individual doctor-patient-communication. Empathy seemed to play a major role for many:

- Yes, well, the interpersonal relationship is sort of important, I guess. Just a little, yes, I really recognized this with my speech therapist. When you’re in a room together for 45 minutes and not just once but oftentimes, then you should better get along with each other.

#### Information needs prior to or during the stay at the hospital

Right before or during hospitalization, patients seemed to want the most amount of information. Surgical procedures were ultimately upcoming and patients wished for detailed information about the surgery as well as their post-surgical care:

- I find it important to know exactly what happens next and also how this will be done. And consequently to understand the whole process.
- I asked a lot of stuff. I guess that was good. Well, maybe you could explain every single step to the patient. That would be pretty nice, I guess. I would rather have too much than too less information.
- It depends on different personalities, of course. Because, you know, I have been very open with regard to this. I was able to listen to everything ‘yes, there will be sawn here and there’ and, well, no problem for me, you know (laughs). But, well, I believe there are many out there who have a lot more fears than I do.

The role of close family members like parents was highlighted by most patients, especially prior to or after orthognathic surgery. Patients wished for more direct information for their relatives and thought this to be very helpful:

- Especially prior to surgery, that’s one of my few points of criticism, the information transfer was not really good. I was alone with the doctor throughout informed consent discussion, without my parents. And I got very contradictory statements, which frightened my parents and me and made us feel insecure.
- They (=parents) were more shocked than I was. They thought it was worse than I thought it was.

#### Source of information

Patients ranked verbal information transfer and direct communication high throughout combined orthodontic-orthognathic surgery treatment, especially in the beginning. For many patients, written information was seen as an adjunct. One patient suggested former patients to be present during one of the first consultations as another information source.

- I appreciate verbal information transfer, because written, because if you get this catalog of all information with all the crazy stuff in it ‘*this* can happen and *this* as well’ like, for example, in anesthesia there are infections and stuff like this which is, like, really unrealistic, but it would be in this catalog and you would have to blend things like these out of your mind.
- Puh, to be honest, I think you get a pretty good sense of everything through talking to the doctors.
- Well, I would suggest both – written and verbal – so that you can read the stuff at home whenever you feel like it. Plus, parents can look at it, too!
- I would have found it extremely helpful, if there had been a former or a current patient present during the first consultations. I think this would have been like communication at eye level and former patients would have possibly told a totally different story than surgeons, for example.

Interestingly, the role of the internet seemed to be a minor one for patients. Most patients stated that refrained from getting information via internet, only one patient talked about finding information on specific websites.

#### Doctor-patient-communication

All patients highlighted the wish for an empathetic way of communication, while they did not seem to be bothered by doctors who show their authority. Yet, they found it beneficial if doctors overcame the inherent communication gradient between specialists and lay-persons.

- Well, what I really believe to be important, is, well, that the doctor should really show interest in the patient and, ehm, well, or if, well, sometimes, there are these patients who do not dare to ask anything. And in this case, the doctor should show interest and accommodate with the patient and well, to sort of take their fears a little bit.
- I think, ehm, to sort of see through the patients’ eyes and proceed on their level.

Some patients noted that even if they understand that *time* for communication might be rare during everyday orthodontic routine, they would have sometimes expected that professionals had taken more time to prepare their patients for every upcoming step of their treatment:

- I believe it gets hard sometimes, to take the time to, let’s say, to go over everything again – what to expect, what the patient needs to expect mentally and, ehm, (pause) I believe that the focus should be led to this, well not the focus, but this should be cultivated, that you know, you kind of get more involved in everything and not just be informed really briefly ‘it’s going to be really tough for you’ and that’s it. You need, ehm, you need to be prepared for specific situations, I think. And this was not really the case.
- I think I only had short conversations, I think. And he (=the doctor) was, he was kind of in a hurry oftentimes. And then we only had like 5 minutes talking time or so and you cannot come up with any decent questions you know, things like these develop over the course of a talk and (frowns) well, yes, I have to say, that I had wishes for more time to talk.

### (B) Professionals’ perspectives

#### Information needs

In contrast to the information needs mentioned by patients themselves, surgeons and orthodontists did not differentiate between varying information needs prior to or during the treatment or even prior to the surgery. They seemed to focus on the information needs before treatment start. Professionals mostly talked about the diversity of individual information needs, varying from one patient to another:

- Well, I usually give my patients the maximum version of information and I wait if they can deal with it or if I should continue with less / undetailed information.
- It totally depends, how much information they (=patients) want to hear and how much information would be good for them. Especially when it comes to surgical procedures. Some patients want to know it all, some patients don’t. But the most important thing is, that they get an overview of their treatment: what, when, how, which appliance and so on. That’s very important.

In addition to that, maxillofacial surgeons and orthodontists talked about their views of parents of orthognathic patients being frequently present during the stay at the hospital. They found the relatives somewhat difficult to deal with during patient’s stay at the hospital:

- Parents are very different, not all are alike. Not all patients are alike either (laughs). Orthognathic patients and their parents are not easy to handle on our care unit. It really depends. The minority of patients and parents deals with the post-surgical situation adequate or prepared. Most of them (=patients) are whiny and family members stand around kind of helpless. But this lasts like 2 days or so and then gets better.

#### Source of information

All surgeons and orthodontists believed that a combination of verbal and written information would be the best way to inform and educate their patients:

- I believe the best way to inform our patients is the combination of written and verbal. Patients can take written information home and re-read it whenever they wish to. Parents might read those flyers or brochures as well.
- Written information does not replace verbal doctor-patient-communcation. You can use it as an adjunct, but I think you have to talk to the patient about everything in detail. That’s important.

The focus-group revealed, that the internet played a major role for patients in general- in the eyes of the questioned professionals. They seemed to be alerted by the potential harms of false or one-sided presentation of orthodontic-orthognathic procedures:

- I frequently get asked by patients what information is valid and good on the internet. And I tell them to get information on the internet, it’s okay. But I also tell them right away, that they should read this kind of information with caution. Well, they should really look at reliable sites. I think by now, you can almost find every detail revolving around orthoganthic surgery on the internet, whole surgical procedures as well.
- Most of them (=patients) already saw a Youtube video about orthognathic surgery, I believe (smiling)
- I see this critically. Because some patients really come to me with pictures from the internet and show me how they want to look at the end. And this is totally unrealistic. It’s not going to happen! And the other day there was this patient who came from another surgeon with a precise simulation of surgery and before- and after-pictures and I just thought ‘never ever is this going to happen that way’. This is very difficult, I think. How do you get these pictures and expectations out of the patient’s mind? I’d rather have totally uninformed patients during first consultation.

#### Doctor-patient-communication

During the focus-group, professionals talked about finding a way to form a trustful relation to their patients from the beginning on. Interdisciplinary teamwork was mentioned repeatedly.

- Well, basically the first consultation is also about taking the patient’s fears and that you set the ground for a trustworthy doctor-patient-relation.
- It’s important that more than one person is present during the first consultation, I mean a surgeon and an orthodontist. Patients can ask both and they can answer their specific questions. Because I experience it a lot that patients say ‘oh, I wanted to ask the surgeon if…’ and then they are disappointed that there’s no surgeon. It makes sense to talk to the patients *together* before treatment starts.

## Discussion

This qualitative study raised the question of adequate patient information in the course of orthodontic-orthognathic surgery treatment. Does ‘one size fit all’ regarding patient information? According to relevant literature and our study results, the answer should be ‘no’. Interestingly, patients talked about their information needs with distinction regarding the specific treatment phase. This aspect did not occur throughout the focus-group of professionals, i.e. orthodontists and surgeons. Although all parties agreed, that patients should have a broad overview prior to treatment start and should receive information “in a nutshell”, only patients themselves claimed the highest demand for professional information before orthognathic surgery and during the stay at the hospital. Within our focus-group, professionals seemed to think that they know what their patients’ information needs were, possibly due to long-lasting experience. Yet, they stressed the importance of individualized information transfer for each patient. Interestingly, some stated, that they „test their patients“, whether they want a lot of information or only the least necessary prior to treatment start, but it remained unclear how they did so. Over time, professionals might develop strategies during everyday orthognathic routine to find out what kind of patient they are dealing with – scared or tough; with high, maybe unrealistic expectations or potentially easy to please – but it is crucial to question those strategies once in a while, alter them, if needed, and ask for psychological assistance when trying to properly assess patient’s psychological traits [21, 24, 25]. As we are usually specialized *dentists*, we might lack detailed *communicative* knowledge, which is especially important when dealing with orthognathic patients. Reflecting the own approach to deliver information is beneficial for our patients as well as for their treatments in specific. Catt et al. investigated the relation between oral health-related quality of life and the quality of communication perceived by orthognathic surgery patients and came to the conclusion, that the better informed patients thought they had been after treatment, the higher was their oral health-related quality of life [26]. In line with our results, patients wanted to be involved in the decision-making process prior to treatment. Patients from our study frequently reported that they found openness and empathy to be crucial for doctor-patient-communication. Although there are still only few studies that look at the correlation between quality of communication and satisfaction and/or oral health-related quality of life in the field of orthognathic surgery, it seems obvious, that ‘being prepared for what comes next’ or ‘being prepared for surgery itself’ is highly important for combined orthodontic-orthognathic patients, especially because this treatment approach mostly includes an *elective* surgery. Cunningham et al. highlighted the aspect of open doctor-patient-communication throughout treatment. Patients should be allowed and even encouraged to ask questions at any point of treatment – or prior to it – and sufficient patient management in orthognathic surgery should include a patient-centered way of communication [10].

Verbal and empathetic doctor-patient-communication was most important for our patients, which was in line with results from relevant research. The factor *time* seemed to play a large role. Some patients wished that medical staff would have taken more time to talk. This should be an alarming signal for an interdisciplinary team. Having these criticisms in mind, every medical staff member of a combined orthodontic-orthognathic team should question their everyday routine. Patient-centered communication, as mentioned above, is a major key to a successful treatment and overall patient satisfaction [10].

Furthermore, written information about combined orthodontic-orthognathic treatment and the potential side-effects is generally a common tool for information transfer [27–30], especially prior to treatment uptake. Patients of our study reported that they found written information mostly beneficial. Written informational material for patients should be generally designed evidence based [30–33]. In addition to that, some researchers declared supplementary visual tools to be additionally helpful in order to inform orthodontic patients [13, 14]. Another way for obtaining information is using the internet. Professionals who were questioned in the course of this study openly addressed their concerns regarding this source of information. On the one hand, some found it to be fine and up-to-date. On the other hand, they questioned the use and clinical significance of such information due to potentially flawed and unfiltered data of personal experiences. Yet, they did not offer a specific way to guide their patients through internet-based information. Social media platforms like YouTube, Twitter and Instagram frequently provide specific and potentially biased information [6, 15, 16, 34]. Patients might be overwhelmed and even frightened by such unfiltered information with a potential to raise unrealistic expectations [35]. Although some patients of our study seemed to reflect about these circumstances, some talked about the chance to get *independent* information by searching the internet. This might be a fallacy, if doctors do not guide patients through internet-based information properly and direct them to validated websites [36]. Interestingly, while professionals agreed on their general experience, that nearly *all* patients who they had met in recent past and who had been in need for orthognathic surgery searched the internet for information prior to their first consultation at the clinic, patients themselves seemed to think differently and did not deliberately mention this potential source of information. This is in line with previous results [27], but then again, international researchers also stated, that the internet *is* a useful tool for orthognathic patients [6, 28, 34, 37]. In the context of our study, one might regard this as a phenomenon which might occur when conducting interviews: The interviewee wants to represent him−/herself in a good light and therefore occasionally modifies statements according to his−/her belief of what the interviewer might seek to hear (social desirability, response bias, acquiescence bias). Because this phenomenon is inherent to some qualitative research methods, we should account for it, but not rank this supposedly bias high.

Some patients mentioned, that close relatives had been essential for them throughout treatment. They felt supported by their family, especially during the stay at the hospital. Within the conducted focus-group, professionals confirmed that patients’ families were frequently present at the hospital, but seemed to have difficulties to deal with the situation properly. Furthermore, patients reported that they had wished for more direct communication from doctor to family member as for instance when side-effects like facial swelling were disconcerting for both patients and family alike. Therefore, close relatives might better be involved in discussions from the beginning. One must keep in mind, that many orthognathic patients are young adults at the age of 18+ years old and might not feel completely grown up and independent yet. On the other hand, their parents might be frightened because they still see their child as such and not an adult. The important factor of interpersonal support has been confirmed by studies which showed that orthodontic-orthognathic surgery patients had a significantly higher number of contacts in their social support network [38]. Social support proofed to be not only important immediately after surgery, but also played a major role regarding general patient satisfaction [10, 39]. The implementation of specific family−/ parent-centered information therefore seems highly necessary.

In our study, some patients mentioned the possibility of being scared away by too much and detailed information. They stated, that if patients were not ‘strong enough’ they could not deal with such information. The individual need for information and the question of how much information might be *too much* information to handle is closely associated with the patients’ expectations of treatment. Cunningham et al. found out that the psychological profiles of patients in need for combined orthodontic-orthognathic surgery differed from a non-affected control group [38]. Ryan et al. made efforts to identify a specific characteristics of orthognathic patients, their expectations and satisfaction. They highlighted the need for pre-treatment psychological screening of patients, together with mental health experts, if needed. As some patients might have unrealistic expectations regarding physical and non-physical changes, professionals must be alert during patients’ first consultations [9]. Mental disorders like the body dysmorphic disorder, where a minor or even imagined defect causes significantly distress [40], have to be thought of when patients report about high expectations, especially regarding non-physical changes [41]. The prevalence of such mental disorders among orthognathic patients has not extensively been described so far, but researchers like Veale et al. estimated it to be 11.2% in the field of orthognathic surgery and 5.2% in orthodontics and cosmetic dentistry [42]. This assumingly high prevalence should be a warning sign for orthodontists as well as for maxillofacial surgeons as they do not regularly have the psychological experience in order to detect and/or treat conspicuous patients. Screening tools like the Dysmorphic Concern Questionnaire might help in this context [43, 44]. Although researchers already reported, that orthodontists might be afraid of referring patients to a mental health professional [24], patients themselves did not seem to be intimidated or have negative feelings about such a referral [21, 45]. Thus, as mentioned above, the need for psychological support within the interdisciplinary team of professionals should not be ignored, but rather addressed [46].

Despite the advantages of the qualitative methods used for this research project, some methodological issues should be raised. A sample size of 16 seems rather small compared to quantitative studies. Yet, as mentioned above, one has to keep in mind, that qualitative studies intentionally and conceptually don’t aim for a large sample size and a statistical numeric generalization, but rather a conceptual generalization [22, 47, 48]. The concept of reflexivity by the researcher is crucial in order to diminish blurring interviewer bias [49]. In this study, the interviewer – and moderator within the focus-group – had been trained and chosen by an interdisciplinary panel of experts, so that potential biasing factors should be regarded as neglectable. Nevertheless, it lies in the very nature of qualitative research that influential personal factors such as gender, age, ethnicity and profession, cannot be avoided. Furthermore, one specific limitation of this study might be seen in the *regional* limitation. However, discussing our results in the light of relevant *international* literature showed, that specific categories or themes were applicable across different regions and nations. This, again, highlights the strengths of qualitative methods searching for conceptual generalization. According to internationally reported unmet needs and expectations of orthognathic patients, an individual-centered way to treat and inform this clientele might still not be fully incorporated during everyday routine and should still be regarded as our main task for the future [2, 6, 10, 34].

## Conclusion

This qualitative study highlights the need for individualized patient information and reveals both met and unmet information needs. Although evidence-based written information is highly necessary for orthognathic patients and their families alike, it cannot replace an empathetic way of direct verbal doctor-patient-communication. It seems crucial to give specific individualized information at different treatment stages, starting with a thoroughly interdisciplinary screening at the very beginning. The internet as a source of information must still be seen with caution and professionals should find a way to guide their patients through it.

## Data Availability

The data underlying this article cannot be shared publicly for no clear reason due to privacy issues of individuals that participated in the study. Yet, the data will be shared on reasonable request to the corresponding author.
